# The potential of thermal imaging as an early predictive biomarker of radiation dermatitis during radiotherapy for head and neck cancer: a prospective study

**DOI:** 10.1186/s12885-025-13734-8

**Published:** 2025-02-20

**Authors:** Ye-In Park, Seo Hee Choi, Min-Seok Cho, Junyoung Son, Changhwan Kim, Min Cheol Han, Hojin Kim, Ho Lee, Dong Wook Kim, Jin Sung Kim, Chae-Seon Hong

**Affiliations:** 1 Department of Radiation Oncology, Yonsei Cancer Center, Heavy Ion Therapy Research Institute, Yonsei University College of Medicine, 50-1 Yonsei-ro, Seodaemun- gu, Seoul, 03722 Korea; 2 Department of Radiation Oncology, Yongin Severance Hospital, Yonsei University College of Medicine, Yongin, Gyeonggi do Korea

**Keywords:** Radiation dermatitis, Skin toxicity, Head and neck cancer, Thermal imaging, Machine learning, Biomarker, Radiotherapy

## Abstract

**Background:**

Predicting radiation dermatitis (RD), a common radiotherapy toxicity, is essential for clinical decision-making regarding toxicity management. This prospective study aimed to develop and validate a machine-learning model to predict the occurrence of grade ≥ 2 RD using thermal imaging in the early stages of radiotherapy in head and neck cancer.

**Methods:**

Thermal images of neck skin surfaces were acquired weekly during radiotherapy. A total of 202 thermal images were used to calculate the difference map of neck skin temperature and analyze to extract thermal imaging features. Changes in imaging features during treatment were assessed in the two RD groups, grade ≥ 2 and grade ≤ 1 RD, classified according to the Common Terminology Criteria for Adverse Events (CTCAE) guidelines. Feature importance analysis was performed to select thermal imaging features correlated with grade ≥ 2 RD. A predictive model for grade ≥ 2 RD occurrence was developed using a machine learning algorithm and cross-validated. Area under the receiver-operating characteristic curve (AUC), precision, and sensitivity were used as evaluation metrics.

**Results:**

Of the 202 thermal images, 54 images taken before the occurrence of grade ≥ 2 RD were used to develop the predictive model. Thermal radiomics features related to the homogeneity of image texture were selected as input features of the machine learning model. The gradient boosting decision tree showed an AUC of 0.84, precision of 0.70, and sensitivity of 0.75 in models trained using thermal features acquired before skin dose < 10 Gy. The support vector machine achieved a mean AUC of 0.71, precision of 0.68, and sensitivity of 0.70 for predicting grade ≥ 2 RD using thermal images obtained in the skin dose range of 10–20 Gy.

**Conclusions:**

Thermal images acquired from patients undergoing radiotherapy for head and neck cancer can be used as an early predictor of grade ≥ 2 RD and may aid in decision support for the management of acute skin toxicity from radiotherapy. However, our results should be interpreted with caution, given the limitations of this study.

**Supplementary Information:**

The online version contains supplementary material available at 10.1186/s12885-025-13734-8.

## Background

Radiation dermatitis (RD) is a common toxic reaction during and after radiotherapy. Most patients undergoing head and neck cancer (HNC) radiotherapy experience RD [1, 2]. Acute RD symptoms may cause itching, pain, and secondary infections, decreasing quality of life and interrupting treatment [3, 4]. Therefore, predicting the occurrence of severe RD before its onset is necessary to make clinical decisions to alleviate the severity of RD symptoms.

Various clinical and dosimetric factors have been identified as predictive factors for the severity of RD [5–8]. Previous studies analyzed the risk of severe RD by focusing on factors identified before radiotherapy without considering parameters directly correlated to the physiological mechanism of RD occurrence. Moreover, predictive models for RD developed using only clinical and dosimetric factors cannot reflect individuals’ diversity in radiosensitivity caused by intrinsic factors such as polymorphism in cytokine genes [9]. It is crucial to consider biomarkers correlating with the cellular response to radiotherapy to enhance the robustness of predictive markers for acute RD.

Thermal imaging is a noninvasive and contactless method that can be potent for assessing asymptomatic toxicity reactions and visible RD symptoms [10–12]. Patients who develop severe RD undergo several radiation-induced physiological skin reactions before visual changes appear on the skin [13]. Several thermal imaging features have been reported as risk factors associated with the severity of RD in patients with breast cancer, which may be due to the inflammatory response in and damage to the skin vasculature [14–17]. However, the association between thermal imaging features and RD grades has not been established in patients with HNC, and to the best of our knowledge, a predictive model for the development of severe RD has not yet been developed. RD symptoms occurring in highly visible areas can be considered as more severe outcomes in patients with HNC who state cosmetic changes as a major concern of cancer treatment [18]. The thermal features that predict the severity of RD should be evaluated separately for patients with HNC, considering differences between treatment strategies and skin physiology.

We aimed to investigate the effectiveness of thermal images obtained during HNC radiotherapy as a predictive biomarker of severe RD before its onset. A thermal imaging feature-based machine learning (ML) model was applied to predict the acute RD in patients with HNC. Predicting severe RD in the early phase of radiotherapy using a thermal camera can aid in patient-specific decisions in skin toxicity management and adaptive radiotherapy.

## Methods

We conducted a prospective study of patients who underwent radiotherapy for HNC between November 2020 and March 2023 at our institution. The study was approved by the Institutional Research Board of Yongin Severance Hospital (approval No. 9-2020-0120) and conducted in accordance with the tenets of the Declaration of Helsinki. Informed consent was obtained from all participants before the commencement of the study. All patients underwent volumetric modulated arc therapy treatment using a 6-MV photon beam. Prescribed doses ranged from 48.3 Gy to 70 Gy in 23–33 fractions. The study included 19 patients, and the patient and treatment characteristics are outlined in Additional Table 1.

### Thermal image acquisition

Thermal images were collected using a FLIR E85 infrared camera (FLIR systems, USA) with a thermal image resolution of 384 × 288. Images were acquired by trained operators from the left and right side of the patient’s neck, considering that RD symptoms can be distributed over a large area of the neck skin. Thermal images were collected from the patients in treatment position after irradiation to ensure the reproducibility of patient posture between images. Thermoplastic masks and clothes that concealed the skin in the irradiation area were removed before imaging. The baseline of skin thermal image was measured on the first day of radiotherapy and repeated routinely once a week during the irradiation schedule and four weeks after the end of treatment. Thermal image was acquired at a distance of about 1 m from each left and right side of patient, and detailed camera position including the shooting height was adjusted for each patient considering the variance in treatment setup. All measurements were performed in the same treatment room to maintain consistency of measurement. The emissivity value in thermal image acquisition was fixed at 0.98, which is acceptable for assessing human skin [19].

### Data processing and feature analysis

**Fig. 1:**
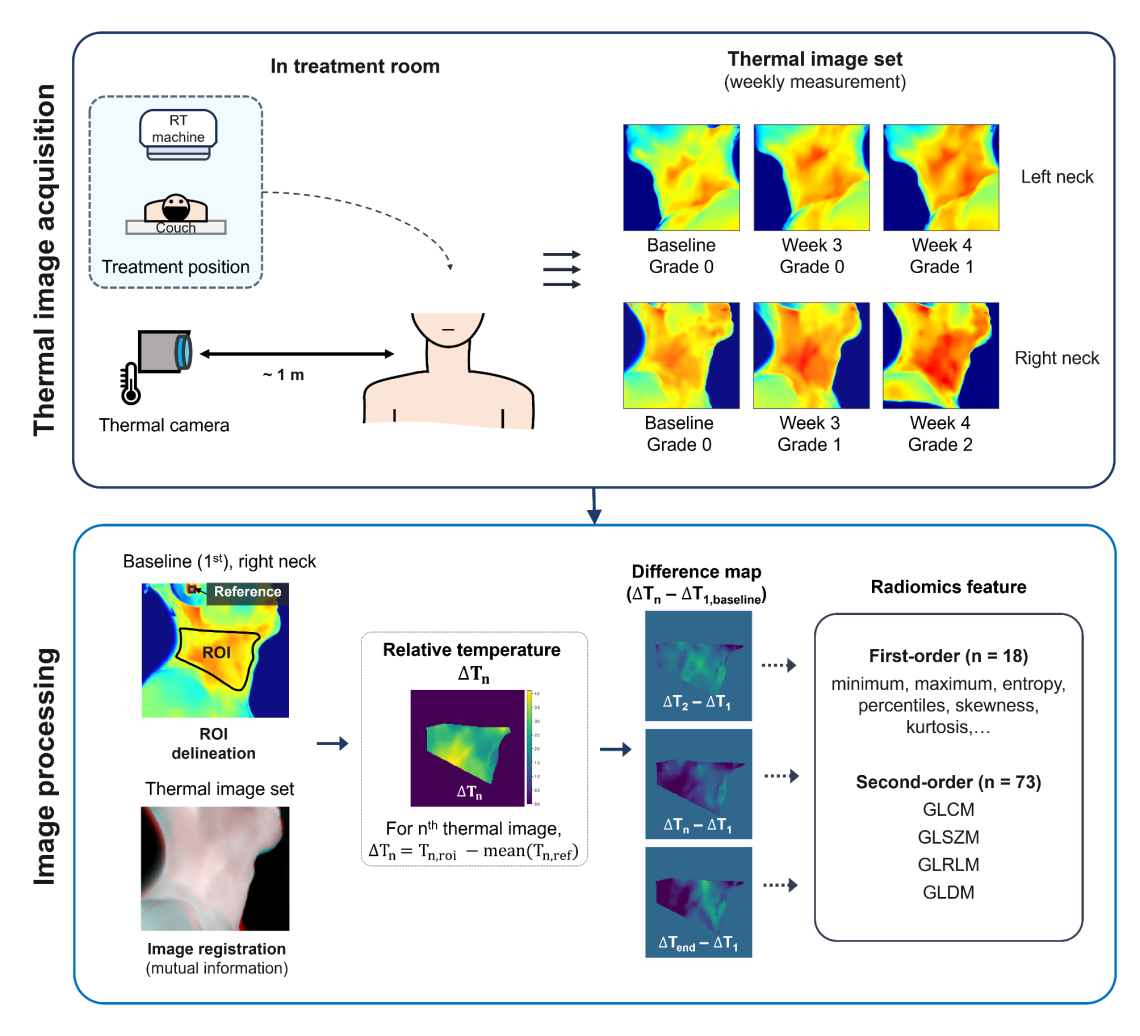
Schematic workflow of thermal image acquisition and image processing. Thermal image acquisition and grading of radiation dermatitis were performed weekly for each left and right neck. ROI, region of interest; GLDM, gray-level dependence matrix; GLSZM, gray-level size zone matrix; GLCM, gray-level co-occurrence matrix; GLRLM, gray-level run length matrix

Data preprocessing was performed to reduce the positional variability between thermal images captured at multiple time points. Thermal images were converted from quantized pixels (jpeg file) into temperature values with a resolution of 0.1 °C using FLIR tools software. Rigid image registration was performed between thermal images acquired from the same camera orientation using mutual information metric and linear interpolation. The thermal image set for each patient included the baseline image and aligned images by rigid registration. Background removal was performed on the thermal image set using Otsu’s threshold method [20]. The region of interest (ROI) for extracting thermal imaging features was defined as the anatomical area from submandibular to supraclavicular and was delineated on the baseline thermal images. Two ROIs were delineated for each patient based on the thermal images acquired from the left and right sides of the neck. Thereafter, the ROI was refined to exclude the skin area blocked out by hair, clothes, and immobilizer, at least once in the thermal image set. Image registration and ROI delineation were performed in MATLAB 2023a (Mathworks Inc., Natick, MA, USA). The thermal image acquisition and the data processing methods were summarized in Fig. 1.

Considering that the absolute temperature value in thermal imaging includes some inaccuracy, pixel values in ROI were converted to a relative temperature scale before the analysis. Since the ear was not included in the irradiation field in all patients, the average temperature at the ear concha in each image was used as a reference value of the relative scale. Relative thermal images were calculated by subtracting the reference value from the ROI temperature map. Difference maps of the relative thermal images between the baseline and each data point were calculated and used to extract the radiomics features. Pixel values in the difference map were discretized with a bin size of 36, in the range of the minimum-maximum value of the entire dataset. A total of 91 radiomic features, consisting of 18 first-order and 73 s-order features, were extracted from pixel values in the difference map of ROIs area using the Pyradiomics library implemented in Python 3.9 (Python Software Foundation, Wilmington, Del, USA). Second-order features consisting of gray-level co-occurrence matrix (GLCM), gray-level size zone matrix (GLSZM), gray-level run length matrix (GLRLM), and gray-level dependence matrix (GLDM) were extracted.

The severity of RD was assessed once a week for each patient simultaneously with thermal imaging collection according to the Common Terminology Criteria for Adverse Events (CTCAE) v.5 guidelines. The severity of RD in thermal images was classified into two groups: CTCAE grade ≥ 2 and grade ≤ 1. Thereafter, a generalized equivalent uniform dose (gEUD) irradiated on the ROI region was computed to assess the change of thermal imaging features concerning radiation dose. The neck area in treatment planning data was defined along the transverse plane with the exact anatomic boundaries used for ROI delineation in thermal imaging, then divided into left and right sides based on the patient center. The dose to each left and right neck skin area was defined as a dose at a 2 mm depth on the patient’s surface automatically delineated from computed tomography using MIM software (MIM Software Inc., Cleveland, OH, USA). The hyperparameters for calculating gEUD from the dose-volume histogram of each neck skin area were set to a/b ratio = 10 Gy and *n* = 0.1 for skin tissue [21, 22]. The cumulative skin dose at each time point of image acquisition was calculated by multiplying the planned gEUD by the ratio of delivered fractions.

### Feature selection and RD prediction

ML-based classification models were trained to predict the occurrence of grade ≥ 2 RD. The endpoint of the predictive model was designed to classify the severity of RD into two groups (grade ≥ 2 vs. grade ≤ 1) based on the highest CTCAE grade reported during RT. Across all patient dataset collected in this study, CTCAE grade ≥ 2 was reported only in images acquired after cumulative gEUD exceeded 30 Gy. Therefore, to train ML models using only features acquired before the occurrence of grade ≥ 2 RD, thermal imaging data acquired after a cumulative gEUD of 30 Gy in all patients was excluded from the analysis. To investigate the most effective time point schedule for predicting the occurrence of grade ≥ 2 RD using thermal images among treatment courses, ML models were trained using a dataset divided into three groups based on the cumulative gEUD range of 0–10 Gy, 10–20 Gy, and 20–30 Gy.

The feature selection process was performed to avoid overfitting the predictive model due to many input features. Predictive factors for modeling were selected by combining the minimum redundancy maximum relevance feature selection (mRMR) method and the least absolute shrinkage and selection operator (LASSO) [23, 24]. First, the mRMR method was used based on mutual information, with a threshold of 10 for the number of selected features. Second, LASSO was conducted using radiomics features that were selected using mRMR, and features with non-zero coefficients were chosen as potential predictors of grade ≥ 2 RD occurrence. The feature selection process was performed using the entire data included in each cumulative gEUD group and repeated 10 times using five-fold cross-validation to ensure robust feature selection. The importance of the radiomics feature was calculated as the frequency at which it was selected as a potential predictor during the feature selection process. Finally, radiomics features with feature importance > 0.5 were applied to train the ML models.

ML experiments were performed for each gEUD range using a support vector machine (SVM), gradient-boosting decision tree (GBDT), and logistic regression (LR). The hyperparameters of the ML models were selected via a grid search algorithm from a parameter space (Additional Table 2). Training and validation of ML models were performed using the 0.632 + bootstrap method with 1000 repetitions to reduce the optimistic bias in prediction performance from small data size [25, 26]. Data resampling with the bootstrap method was performed on a patient-by-patient basis, and thermal radiomics features acquired from different sides of the neck were considered independent data. The prediction performance of the trained models was evaluated using the area under the receiver operating characteristic (ROC) curve (AUC), precision, and sensitivity.

## Results

Thermal images were collected from patients enrolled in this study at 101 time points from a total of 19 patients (mean age 58.8 years). Each patient’s average number of thermal image acquisitions was 5.9 (range: 4–7). Among the thermal images, 202 acquired from the left and right sides of the patients were analyzed. Of the 19 patients, 14 (73.7%) presented dry desquamation and grade ≥ 2 RD during their treatment duration. The number of patients with grade ≥ 2 RD observed in the left and right sides of the neck was 13 and 12, respectively. Of the thermal images from patients with grade ≤ 1 RD as an endpoint, 10 were acquired in the cumulative gEUD range of 0–10 Gy, 19 were in 10–20 Gy, and 13 were in 20–30 Gy. For patients with grade ≥ 2 RD occurrence during radiotherapy, thermal images acquired in cumulative gEUD range of 0–10 Gy, 10–20 Gy, and 20–30 Gy were 9, 35, and 31, respectively.

The results of ML models developed using thermal imaging features with different gEUD ranges are summarized in Table 1. The overall performance in predicting RD was highest when ML models were trained using a thermal radiomics feature set extracted in the cumulative gEUD range of 10–20 Gy. The effective timing of thermal imaging acquisition for predicting the grade ≥ 2 RD occurrence was selected as cumulative gEUD range 10–20 Gy, corresponding to the first two weeks from the first day of radiotherapy in this study.

**Table 1:** Predictive performance of machine learning models trained using three different data groups

	Input features	Models	AUC	Precision	Sensitivity
gEUD 0–10 Gy (*n* = 19)	GLRLM gray-level non uniformity, NGTDM contrast	SVM	0.74	0.11	0.69
		GBDT	0.84	0.70	0.75
		LR	0.72	0.56	0.60
gEUD 10–20 Gy (*n* = 54)	GLDM dependence variance normalized, GLSZM large area low gray-level emphasis, GLCM inverse difference	SVM	0.71	0.68	0.70
		GBDT	0.75	0.79	0.56
		LR	0.60	0.64	0.52
gEUD 20–30 Gy (*n* = 44)	GLSZM size zone non uniformity	SVM	0.57	0.79	0.92
		GBDT	0.57	0.78	0.83
		LR	0.56	0.78	0.45

Performance is reported with respect to the evaluation metrics in 0.632 + bootstrap method

*Abbreviations*: gEUD, generalized equivalent uniform dose; SVM, support vector machine; GBDT, gradient-boosting decision tree; LR, logistic regression; AUC, area under the receiver operating characteristic curve; GLDM, gray-level dependence matrix; GLSZM, gray-level size zone matrix; GLCM, gray-level co-occurrence matrix; GLRLM, gray-level run length matrix

**Fig. 2:**
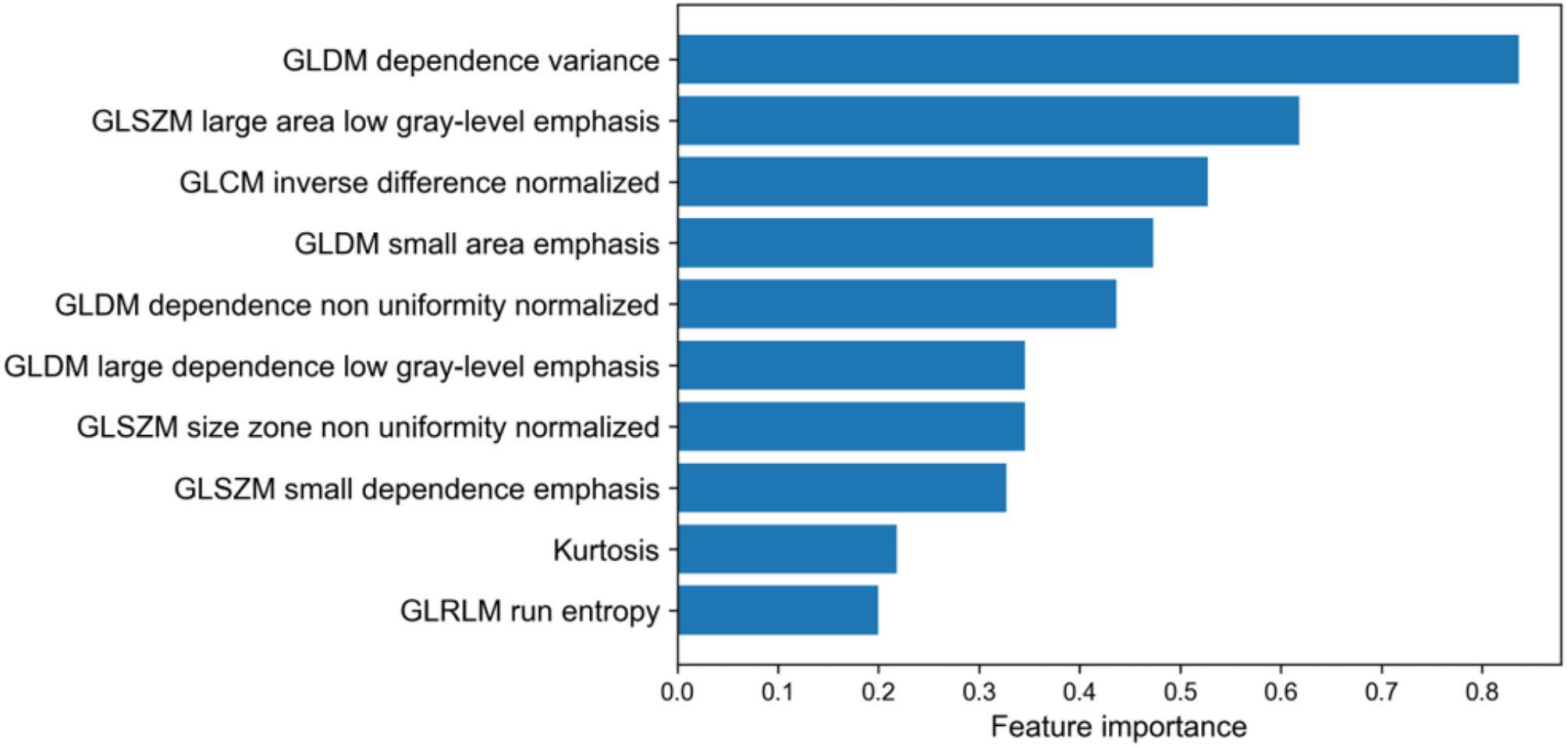
Feature importance identified by the feature selection method using five-fold cross-validation with 10 repetitions. GLDM, gray-level dependence matrix; GLSZM, gray-level size zone matrix; GLCM, gray-level co-occurrence matrix; GLRLM, gray-level run length matrix; Idn, inverse difference normalized

**Fig. 3:**
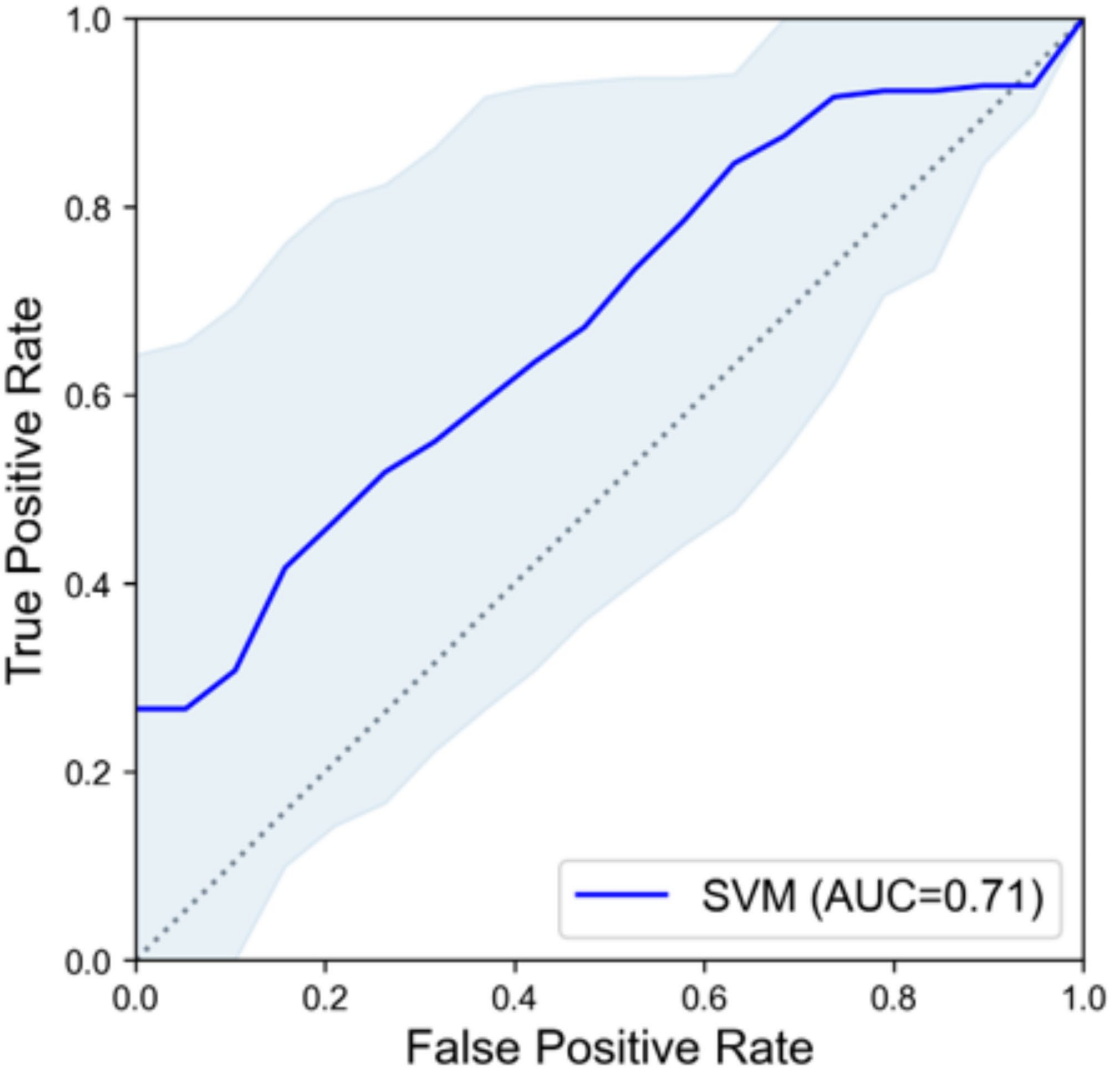
Receiver operating characteristic curve of the support vector machine (SVM) model. This was based on a mean value of 0.632 + bootstrap method. The shadowed area is the 95% confidence interval. The SVM model was trained to predict the occurrence of grade ≥ 2 radiation dermatitis using thermal imaging features obtained in skin generalized equivalent dose (gEUD) of 10–20 Gy, and the area under the curve (AUC) value was 0.71

A total of 54 thermal images obtained in the selected range were applied for feature selection and model development. Figure 2 shows the feature importance of the first ten most selected features in the repetition of the feature selection method. Three thermal imaging features, GLDM dependence variance, GLSZM large area low gray-level emphasis (LALGLE), and GLCM inverse difference normalized (Idn), were selected as input features in the cumulative gEUD range 10–20 Gy. In the cumulative gEUD range, 10–20 Gy, the GBDT model indicated the highest AUC of 0.75, and the SVM model showed > 0.6 in all metrics and was selected as representative. The SVM model performance in the 0.632 + bootstrap method achieved an AUC of 0.71, precision of 0.68, and sensitivity of 0.70, and the ROC curve is shown in Fig. 3 and Additional Fig. 1. Feature selection and evaluation metrics of ML models in other cumulative gEUD ranges are summarized in Table 1. 

## Discussion

Predicting the occurrence of radiation-induced skin toxicity in the early phases of radiotherapy can be beneficial for making clinical decisions regarding skin management during the fractionation schedule. The aim of this study was to investigate the validity of thermal imaging features as predictors of grade ≥ 2 RD in patients with HNC. We acquired patients’ skin thermal images weekly and examined the differences in skin thermal imaging features depending on RD grades. The predictive model for RD was trained and validated based on thermal imaging features collected before the occurrence of grade ≥ 2 RD and more than half of ML models achieved an AUC > 0.7. Therefore, thermal images taken from the neck surface may be used to develop a predictive model for grade ≥ 2 RD in patients receiving head and neck radiotherapy.

Acute RD is a common toxic reaction in HNC patients receiving radiotherapy and occurs within the irradiated skin in many cases. Repeated exposure to ionizing radiation elevates the expression of cytokines that promote inflammation and local immunity at the target site [27]. Pro-inflammatory cytokine, vascular injury, and other complex physiologic changes can cause the dilation of blood vessels, which leads to erythema and other dermatitis symptoms [28, 29]. A significant correlation between RD grade and cutaneous blood flow has been demonstrated in patient studies [13, 30]. Since blood perfusion plays a role in skin temperature regulation, measuring skin temperature can be used to monitor the inflammatory response before RD symptoms appear [16, 31]. Our results indicate that thermal imaging features can be used as biomarkers of grade ≥ 2 RD in patients with HNC.

Changes in skin temperature may be effective in monitoring the physiological reactions directly associated with the occurrence of grade ≥ 2 RD, independent of tumor type and treatment technique. The correlation between RD grade and change in thermal imaging features has been investigated for patients receiving breast radiotherapy [15, 32]. Previous studies in breast cancer patients reported that ML models utilizing thermal radiomics features can predict the RD grade with a high AUC value > 0.9. The AUC difference with the model for breast RD may be due to the small number of datasets and the difference in treatment technique. This study focused on assessing the feasibility of thermal radiomics features as biomarkers of grade ≥ 2 RD in HNC. Thus, further investigation is needed to improve the predictive performance of ML models. Exploring other ML models and utilizing multimodal datasets, including thermal and clinical features, can enhance the precision and reliability of the predictive model.

This study is the first to develop a predictive model for RD in HNC patients by monitoring the skin temperature during radiotherapy. A previous study using thermal images for detecting oral mucositis reported no statistical significance between RD grade and change in average skin temperature [10]. The difference in the feasibility of thermal imaging features in predicting RD may be because the previous study analyzed thermal imaging features acquired from patients at risk of oral mucosa. Both RD and oral mucosa involve thermal changes from inflammatory responses. Therefore, the significance of thermal imaging features in predicting RD should be investigated in patients who develop RD as a major radiotherapy toxicity.

The results in Table 1 showed that ML models could predict the occurrence of grade ≥ 2 RD using radiomics features extracted from the change of skin temperature within cumulative gEUD 20 Gy. ML models using cumulative gEUD 10–20 Gy features showed good performance in AUC and other evaluation metrics. If the dose irradiated to the patient’s skin was high and similar to the prescribed dose to target site, the cumulative gEUD in ROI will exceed 20 Gy after 10 fractions, corresponding to the two weeks after treatment. Therefore, thermal radiomics features in cumulative gEUD < 20 Gy can be obtained in all patients by acquiring thermal images within two weeks. Thermal image acquisition at two time points, on the first day of radiotherapy and once within the two weeks of treatment, can serve as effective thermal characteristics to predict grade ≥ 2 RD in patients with HNC. Since the reasons for the poor performance of ML models in the cumulative gEUD range of 20–30 Gy is unclear, it should be investigated in a large data set.

Most thermal radiomics features selected as predictors of the severity of RD were related to temperature homogeneity. GLRLM gray-level nonuniformity indicates the homogeneity in intensity value. GLDM dependence variance may be associated with texture homogeneity because GLDM quantifies the number of connected pixels within a distance dependent on the central pixel’s gray value [33]. The texture feature GLSZM-LALGLE measures the joint distribution of large-sized zones with lower gray-level values, which describes the preponderance of large areas with low-density pixels [34]. GLCM Idn indicates the local homogeneity within ROIs. A similar finding in the relationship between grade ≥ 2 RD and skin temperature heterogeneity has been demonstrated in breast radiotherapy [17, 32]. Further investigations are required to determine whether texture variation in the skin thermal map is a predictive factor for RD, independent of the clinical and imaging parameters such as image resolution.

The proposed approach to predict RD using thermal images can be beneficial in clinics when deciding on early supportive care interventions and planning strategies to decrease the risk of severe RD in patients with HNC. Severe RD symptoms in patients undergoing head and neck radiotherapy can show inhomogeneous distribution across the skin adjacent to the target [35, 36]. Therefore, measuring thermal images from a single fixed location may not be sufficient to investigate the thermal imaging features that strongly correlate with the occurrence of severe RD in patients with HNC. Our predictive model was trained and evaluated using thermal images of the left and right sides of the patients’ necks as independent data, which allows the prediction of the occurrence of grade 2 or higher RD and the estimation of its location. Moreover, obtaining multiple images from each patient helps increase the data that can be used for training the ML model. Since image acquisition was performed in the treatment room with an average of less than two minutes, the proposed procedure may be easily implemented in clinics.

Our study has several limitations. First, the sample size was small. The severity of RD was categorized into only two classes, grade ≤ 1 and grade ≥ 2, due to the insufficient sample size for each CTCAE grade. Moreover, using a small sample size to train ML models can lead to over-optimistic performance. Although the 0.632 + bootstrap-based optimism correction method was performed to reduce the risk of overfitting, our models should be validated in a larger patient cohort encompassing different treatment modalities. Therefore, our findings need to be interpreted and generalized with caution, given the limited number of patients from a single institution and the risk of bias and overfitting of the models. Future work should integrate these findings into predictive model development to emphasize external validation and different treatment modalities and prevent overfitting when translating machine learning models into clinical practice. Second, thermal image acquisition and ROI delineation were performed manually, which can be labor-intensive to implement in clinics and can include bias and variability in the process. The development of a data-processing module utilizing both thermal and color images improves the clinical usability of the proposed framework. Introducing an automated ROI delineation system based on anatomical structures using color images will help increase data reproducibility in future investigations.

## Conclusions

Thermal images may be used to develop a predictive model for RD early during radiotherapy in patients with HNC. This study demonstrated that early changes in thermal imaging features of the neck surface of patients have the potential to be a discriminator associated with the occurrence of grade ≥ 2 RD during treatment. The proposed framework using the thermal camera can provide information regarding the risk of severe RD lateral to the neck surface, which can be beneficial as a tool for patient-specific decision-making regarding RD management during radiotherapy. However, our results should be interpreted with caution, given the limitations of this study. Further studies with more patient data will help clarify the correlation between severe RD and variations in the thermal imaging features of the neck skin surface.

## Electronic supplementary material

Below is the link to the electronic supplementary material.

**Supplementary Material 1**: Additional Fig. 1 and Additional Table 1, and 2. **Additional Table 1**: Patient characteristics. **Additional Table 2**: Grid search setup for hyperparameter tuning and **Additional Fig. 1**: Receiver operating characteristic (ROC) curves of different prediction models.

## Data Availability

Research data are stored in an institutional repository and will be shared upon request to the corresponding author.

## Abbreviations

AUC: Area under the ROC curve
CTCAE: Common Terminology Criteria for Adverse Events
GBDT: Gradient-boosting decision tree
gEUD: Generalized equivalent uniform dose
GLCM: Gray-level co-occurrence matrix
GLSZM: Gray-level size zone matrix
GLDM: Gray-level dependence matrix
GLRLM: Gray-level run-length matrix
HNC: Head and neck cancer
Idn: Inverse difference normalized
LALGLE: Large-area low gray-level emphasis
LASSO: Least absolute shrinkage and selection operator
LR: Logistic regression
ML: Machine learning
mRMR: Maximum relevance feature selection
RD: Radiation dermatitis
ROC: Receiver operating characteristic
ROI: Region of interest
SVM: Support vector machine
