# Supplementary material for: The potential of thermal imaging as an early predictive biomarker of radiation dermatitis during radiotherapy for head and neck cancer: a prospective study
Source: BMC Cancer. 2025 Feb 20;25:309. doi: 10.1186/s12885-025-13734-8 (PMC11844184; doi:10.1186/s12885-025-13734-8)
Supplement: Supplementary file 1 — Supplementary Material 1: Additional Fig. 1 and Additional Table 1, and 2. Additional Table 1: Patient characteristics. Additional Table 2: Grid search setup for hyperparameter tuning and Additional Fig. 1: Receiver operating characteristic (ROC) curves of different prediction models. [file 12885_2025_13734_MOESM1_ESM.docx]

**Supplementary data**

**Additional Table 1** Patient characteristics

| **Patient number** | **Age**  **(years)** | **Sex** | **Prescription**  **dose (cGy)** | **Total**  **fractions** | **Tumor site** | **Chemotherapy** | **Toxicity^a^ (CTCAE)** |
| --- | --- | --- | --- | --- | --- | --- | --- |
| Pt 1 | 64 | Male | 6360 | 30 | Hypopharynx | Yes | G1 |
| Pt 2 | 64 | Male | 6300 | 30 | Oropharynx | No | G2 |
| Pt 3 | 41 | Male | 6600 | 30 | Parotid gland  /salivary | No | G2 |
| Pt 4 | 44 | Female | 6300 | 30 | Oral cavity | Yes | G3 |
| Pt 5 | 66 | Female | 6300 | 30 | Oropharynx | No | G2 |
| Pt 6 | 57 | Male | 6996 | 33 | Oropharynx | Yes | G1 |
| Pt 7 | 49 | Male | 6996 | 33 | Oropharynx | Yes | G2 |
| Pt 8 | 62 | Male | 6300 | 30 | Oral Cavity | Yes | G2 |
| Pt 9 | 58 | Male | 6000 | 30 | Oral Cavity | Yes | G2 |
| Pt 10 | 50 | Male | 6600 | 30 | Oropharynx | Yes | G3 |
| Pt 11 | 62 | Female | 6300 | 30 | Hypopharynx | Yes | G2 |
| Pt 12 | 77 | Male | 6600 | 30 | Larynx | No | G3 |
| Pt 13 | 59 | Male | 6300 | 30 | Hypopharynx | Yes | G2 |
| Pt 14 | 48 | Female | 6300 | 30 | Oral cavity | Yes | G2 |
| Pt 15 | 63 | Male | 6600 | 33 | Larynx | Yes | G2 |
| Pt 16 | 86 | Male | 4500 | 15 | Other | Yes | G1 |
| Pt 17 | 59 | Male | 6300 | 30 | Larynx | Yes | G2 |
| Pt 18 | 72 | Male | 6360 | 30 | Parotid gland  /salivary | No | G1 |
| Pt 19 | 67 | Male | 6300 | 28 | Larynx | No | G1 |

*Abbreviations*: CTCAE: Common Terminology Criteria for Adverse Events; ^a^ Radiation dermatitis was graded according to the CTCAE v5.0

**Additional Table 2** Grid search setup for hyperparameter tuning

|  | **SVM** | | **GBDT** | | | **LR** |
| --- | --- | --- | --- | --- | --- | --- |
| **Hyperparameters** | **C** | **γ** | **Number of tree** | **Maximum depth** | **Learning rate** | **C** |
| Parameter space | [0.001,0.01,0.1,  1,10,100] | | 20,30,50,80,100 | 1,2,3 | [0.001,0.01,0.05, 0.1,0.5,1] | [0.001,0.01,0.1,  1,10,100] |
| Selected parameter | 10 | 1 | 100 | 2 | 0.01 | 1 |

*Abbreviations*: SVM, support vector machine; GBDT, gradient-boosting decision tree; LR, logistic regression


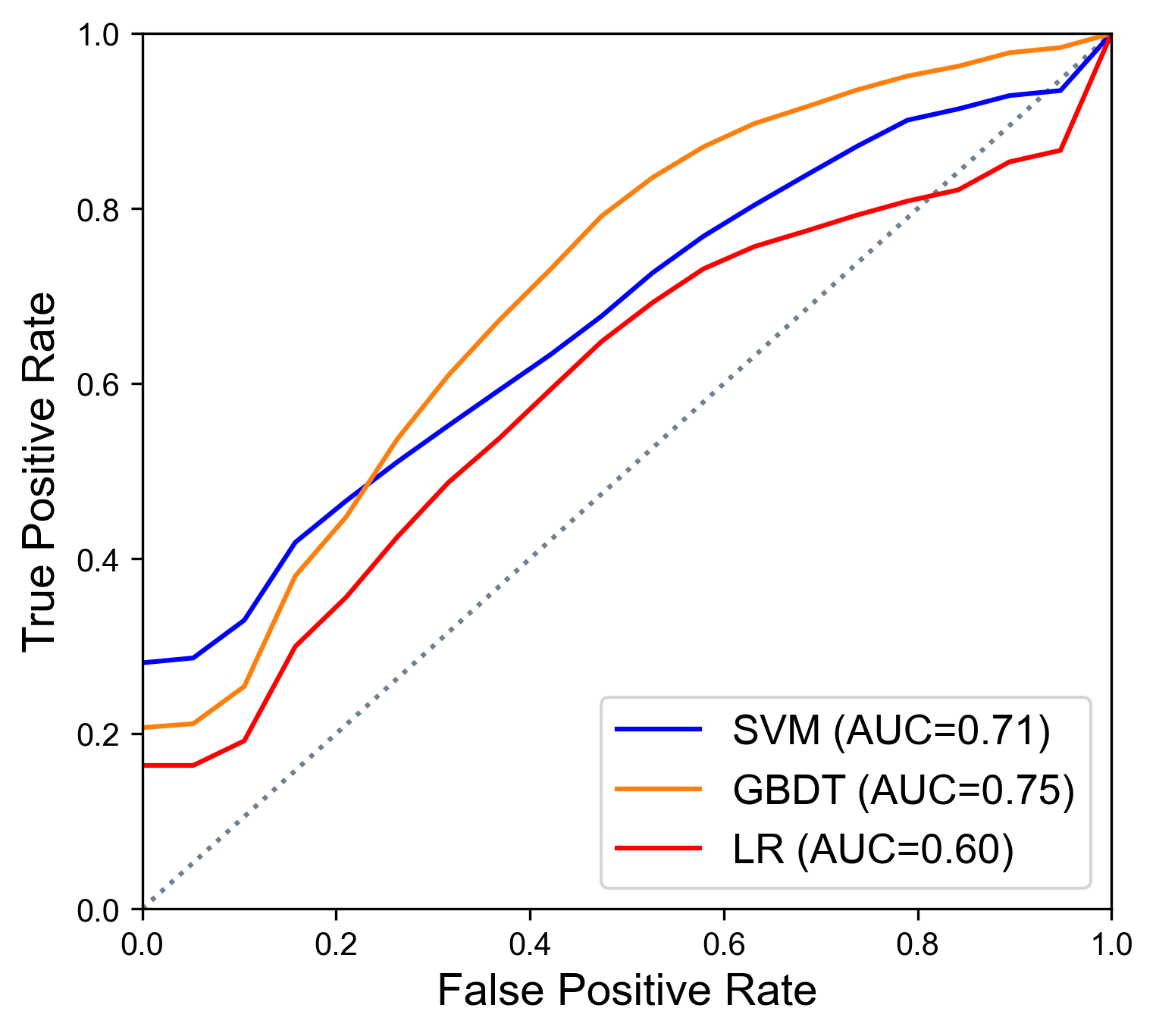


**Additional Figure 1** Receiver operating characteristic (ROC) curves of different prediction models

AUC, area under ROC curve; SVM, support vector machine; GBDT, gradient-boosting decision tree; LR, logistic regression.
